# Enhanced antiviral immunity and dampened inflammation in llama lymph nodes upon MERS-CoV sensing: bridging innate and adaptive cellular immune responses in camelid reservoirs

**DOI:** 10.3389/fimmu.2023.1205080

**Published:** 2023-06-14

**Authors:** Jordi Rodon, Nigeer Te, Joaquim Segalés, Júlia Vergara-Alert, Albert Bensaid

**Affiliations:** ^1^ Unitat Mixta d’Investigació IRTA-UAB en Sanitat Animal, Centre de Recerca en Sanitat Animal (CReSA), Catalonia, Spain; ^2^ IRTA, Programa de Sanitat Animal, Centre de Recerca en Sanitat Animal (CReSA), Catalonia, Spain; ^3^ Departament de Sanitat i Anatomia Animals, Facultat de Veterinaria, Universitat Autònoma de Barcelona (UAB), Catalonia, Spain

**Keywords:** camelids, cytokines, immune responses, llama, lymph node, lymphocyte, Middle East respiratory syndrome coronavirus (MERS-CoV)

## Abstract

Middle East respiratory syndrome coronavirus (MERS-CoV) infection can cause fatal pulmonary inflammatory disease in humans. Contrarily, camelids and bats are the main reservoir hosts, tolerant for MERS-CoV replication without suffering clinical disease. Here, we isolated cervical lymph node (LN) cells from MERS-CoV convalescent llamas and pulsed them with two different viral strains (clades B and C). Viral replication was not supported in LN, but a cellular immune response was mounted. Reminiscent Th1 responses (IFN-γ, IL-2, IL-12) were elicited upon MERS-CoV sensing, accompanied by a marked and transient peak of antiviral responses (type I IFNs, IFN-λ3, ISGs, PRRs and TFs). Importantly, expression of inflammatory cytokines (TNF-α, IL-1β, IL-6, IL-8) or inflammasome components (NLRP3, CASP1, PYCARD) was dampened. The role of IFN-λ3 to counterbalance inflammatory processes and bridge innate and adaptive immune responses in camelid species is discussed. Our findings shed light into key mechanisms on how reservoir species control MERS-CoV in the absence of clinical disease.

## Introduction

The Middle East respiratory syndrome coronavirus (MERS-CoV) can cause severe pneumonia and is associated with a high case-fatality rate in humans (1). Currently, clade B strains have a high incidence in the Arabian Peninsula, while clade C strains, restricted to Africa, are not causing outbreaks despite that reactive virus-specific T-cells were found in humans (2). MERS-CoV was reported to abortively infect human T cells *in vitro* and concomitantly induce apoptotic pathways (3), which might explain the severe lymphopenia commonly reported in MERS patients (4, 5). Altogether, these findings could lead to aberrant or delayed induction of antiviral T cell responses, as observed in acute phase patients (6–9), and contribute to the high pathogenicity of MERS-CoV. Even so, recovered patients mount effective T cell responses that play a major role in the outcome of MERS. Remarkably, virus-specific CD8^+^ T cell responses were also developed by all survivors studied, including those with undetectable antibody responses (10), suggesting that convalescent patients would trigger early protective immune responses upon a subsequent MERS-CoV infection. Moreover, the crucial role of T cell responses to counteract MERS-CoV infection was quickly unravelled in animal model studies. Contrary to B-cell deficient and control animals, viral persistence was reported in the lungs of T-cell deficient mice (11). Thus, development of robust and functional T cell responses is required to fully achieve MERS-CoV clearance.

MERS-CoV is transmitted to humans by dromedary camels, the main reservoir host (12–14), although other camelid species are also susceptible to viral infection (15–19). These species only develop a subclinical infection, which typically show upper respiratory tract replication and abundant MERS-CoV shedding (14, 20, 21). Camelids elicit strong innate immune responses with dampened inflammation at the mucosal level (22, 23) similar to those described in bat cells (10, 24, 25). Indeed, bats are tolerant to many viruses including MERS-CoV-like viruses (26) and can be experimentally infected with MERS-CoV without suffering from disease (27). These two reservoir species can be reinfected (26, 28), allowing viral maintenance and eventual spread. Therefore, adaptive immune responses must be important determinants of host disease resistance but do not interrupt viral maintenance within these animal populations. Indeed, protective humoral immune responses against MERS-CoV are known to occur in camelids after natural and experimental infection (15–20, 29). Efficient antigen presentation in draining lymph nodes (LN) is essential to ensure successful induction of specific T and B cell adaptive immune responses. Previous experimental studies have shown the presence of infectious MERS-CoV in LN of dromedary camels (20, 21). Moreover, in llamas, abundant nucleoprotein antigen was observed within dendritic-like cells in cervical LNs at 4 days post experimental inoculation (dpi) and MERS-CoV RNA persisted until 24 dpi (30). Although no tissue damage was observed, it is unclear whether the virus could replicate in these lymphoid organs. In the present study, we mimicked a secondary exposure to MERS-CoV clade B and C strains *in vitro* in cervical LN cells from previously inoculated llamas to investigate viral replication and cellular immune responses at the transcriptional level.

## Methods

### Animal welfare and ethics

Animal and laboratory experiments with MERS-CoV were performed at the biosafety level-3 (BSL-3) facilities of the Biocontainment Unit of IRTA-CReSA (Barcelona, Spain). Animal samples used in this work were obtained during necropsy procedures of previous studies (31, 32), approved by the Ethical and Animal Welfare Committee of IRTA (CEEA-IRTA) and by the Ethical Commission of Animal Experimentation of the Autonomous Government of Catalonia (file No. CEA-OH/10942/1).

### Cell culture and MERS-CoV

Vero E6 cells (CRL-1586, ATCC, USA) were cultured in Dulbecco’s modified Eagle medium (DMEM; Lonza, Switzerland) supplemented with 5% FCS, 100 U/mL penicillin, 100 µg/mL streptomycin, and 2 mM glutamine.

A passage-3 MERS-CoV Qatar15/2015 strain (clade B; GenBank accession no. MK280984.2) stock and a passage 6 of the MERS-CoV Egypt/2013 strain (clade C; GenBank accession no. KJ477103) were prepared as previously described (31, 32). Infectious virus titers were assayed in Vero E6 cells and determined by the dilution causing 50% of cytopathic effect in cell cultures (50% tissue culture infectious dose endpoint, TCID50).

### Animal infection and sampling

Two animals were experimentally infected with the Qatar15/2015 strain (31) and two other llamas with the Egypt/2013 strain (32). Infection was monitored for 3 weeks. Nasal swabs samples were obtained daily until 15 days post inoculation (dpi), plus at 17 and 22 dpi. Sera samples were obtained before MERS-CoV challenge and at 7, 14 and 22 dpi, when animals were euthanized and necropsied.

### Cervical lymph node cell isolation

At necropsy day, cervical LNs were collected in Roswell Park Memorial Institute 1640 medium (RPMI, Lonza, Switzerland) supplemented with 100 U/mL penicillin, 100 µg/mL streptomycin, 2 mM glutamine and 10% FCS (all ThermoFisher Scientific, USA) and kept at 4°C until transferred to the lab. LNs were mechanically disaggregated with a sterile blade. Cells were filtered through 70 µm strainers (Corning, USA) and concentrated by centrifugation. Red blood cells were removed using ACK lysing buffer (ThermoFisher Scientific, USA), according to the manufacturer’s instructions. LN cells were resuspended and cultured in RPMI supplemented with 10% fetal calf serum (FCS; EuroClone, Italy), 100 U/mL penicillin, 100 µg/mL streptomycin, 2 mM glutamine and 5×10^-5^ M β-mercaptoethanol (Sigma-Aldrich, USA).

### MERS-CoV exposure to lymph node cells

After isolation, LN cells were cultured in triplicates. One million cells were seeded onto 24-well plates in 1 mL final volume of cell culture medium alone (mock) or containing MERS-CoV Qatar15/2015 or Egypt/2013 strain (MOI of 0.1) and cultured for 48 h at 37°C and 5% CO_2_. Culture supernatants and cells were collected at 0, 24 and 48 hours post-exposure (hpe). Additional fresh control LN cells were also collected prior culture.

### Viral and cellular RNA extraction

Viral RNA was extracted from supernatant samples using the IndiMag pathogen kit (Indical Biosciences, Germany) and a Biosprint 96 workstation (Qiagen, Germany), according to the manufacturer’s instructions. Total RNA was extracted from llama LN cells using the Direct-zol RNA Miniprep (Zymo research, USA), following the manufacturer’s protocol. After RNA extraction, an additional HL-dsDNase treatment using the Heat&Run gDNA removal kit (ArcticZymes Technologies, Norway) was performed according to the manufacturer’s protocol to completely remove the reminiscent genomic DNA. Finally, 1 U/µL RNase inhibitors (Invitrogen, Life Technologies, Waltham, USA) were added. Samples were stored at -75°C until further analyses. The purity and quantity of the extracted RNA were assessed using a BioDrop *µLITE* Spectrophotometer (BioDrop Ltd, UK). A260:A280 ratio ranged from 1.6 to 1.8.

### MERS-CoV RNA detection by RT-qPCR

Viral genomic RNA was detected in culture supernatant by performing the UpE RT-qPCR assay (33), with minor modifications as previously described (15, 29, 34). Samples with a quantification cycle (Cq) value ≤40 were considered positive.

### cDNA synthesis

One hundred and ten ng of total RNA were converted to cDNA in a final volume of 10 μL using the PrimeScript RT reagent Kit (Takara, Japan), by a combination of oligo-dT and random hexamers, following the manufacturer’s instructions. cDNA samples were stored at -75°C until subsequent use.

### Fluidigm biomark microfluidic RT-qPCR

Transcription of cytokines and immune-related genes were quantified using a previously validated protocol to study camelid immune responses (22, 35). A Fluidigm Biomark microfluidic RT-qPCR assay was used to quantify immune-gene expression of LN cell samples. In addition, specific primers for the quantification of MERS-CoV subgenomic RNA (M mRNA) were added to the assay (36). Amplification reactions were coupled with Tm analyses to ensure that specific amplifications occurred. Non-template controls were also included in the assays.

### Relative quantification and data analysis

Gene expression analyses were performed as previously described (35). Briefly, data were collected with the Fluidigm Real-Time PCR Analysis software 4.1.3 (Fluidigm Corporation, USA) and analyzed with the DAG expression software 1.0.5.6 (37). The relative standard curve method (see Applied Biosystems user bulletin #2) was applied to compare gene expression levels of LN cells cultured in different conditions against those of freshly prepared LN cells, using multiple reference gene normalization (*GAPDH*, *HPRT1* and *UbC*). Relative expression of *IFN-λ1* and *IFN-λ3* was calculated according to the 2^-ΔΔCT^ method (38), using the same normalizer genes, since expression levels of these genes in control samples were too low to generate standard curves. Relative expression of each gene in a particular sample was expressed in mean fold-change values (Fc) and are shown in **Supplementary Table 1**.

The unpaired t-test was used to statistically compare the relative expression levels of genes from LN cells exposed to MERS-CoV Qatar15/2015, Egypt/2013 and those of cells cultured in media only. All statistical analyses were performed using GraphPad Prism 9.3.1 (GraphPad Software, USA). Differences were considered significant at *p*-values < 0.05.

## Results

Four llamas were primed by experimental inoculation with MERS-CoV Qatar15/2015 (*n = 2)* or Egypt/2013 (*n = 2)* strains, causing productive infection resolved at 8 to 9 dpi. None of the inoculated llamas displayed clinical signs along the study. Genomic and subgenomic viral RNA were detected for both strains at similar levels in nasal swabs (**Supplementary Figure 1A, B**). Thus, llamas shed high titers of infectious virus independently of the strain causing infection (**Supplementary Figure 1C**). Animals from both groups seroconverted to MERS-CoV with similar levels of nAbs that were detected from 2 weeks after infection onwards (**Supplementary Figure 1D**). Overall, llamas followed similar trends in viral shedding and development of humoral responses regardless of the MERS-CoV strain inoculated.

**Figure 1:**
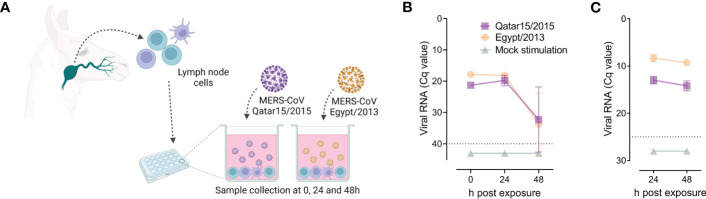
Susceptibility of llama lymph node (LN) cells to MERS-CoV infection. Solid lines indicate mean values and light represent standard deviation intervals. At 22 days after intranasal inoculation of MERS-CoV of llamas, primed LN cells were isolated and pulsed with the same MERS-CoV strain used for animal inoculation, as represented in panel **(A)**. Panels **(B)** and **(C)** display data from llama LN cells seeded in triplicates and exposed for 24 and 48 h to MERS-CoV Qatar15/2015 (purple rectangles), Egypt/2013 (orange circles) or cultured in media only (green triangles). Mean values (± SD) of genomic viral RNA detection in culture supernatants **(B)** and cell-associated viral RNA **(C)** were monitored throughout the study. Grey dashed lines depict the detection limits of the assays. Cq, quantification cycle; MERS-CoV, Middle East respiratory syndrome coronavirus.

Three weeks after infection, llama cervical LN were collected and their cells cultured in the presence of MERS-CoV for 0, 24 and 48 h, as schematically represented in **Figure 1A**. Cells were exposed to the same MERS-CoV strain used for priming. We monitored viral titres in culture supernatants and seeded cells. Importantly, MERS-CoV was not found in cervical LN cells at 22 dpi, as evidenced by the absence of viral RNA in mock-treated cells (**Figure 1B, C**). Independently of the strain used to pulse cells, viral loads in supernatant samples decreased over time, as determined by RT-qPCR for genomic RNA detection (**Figure 1B**). Also, microfluidic RT-qPCR results indicated that cell-associated MERS-CoV RNA declined (**Figure 1C**). Therefore, cervical LN cells of llama did not support MERS-CoV replication at least *in vitro*.

**Figure 2:**
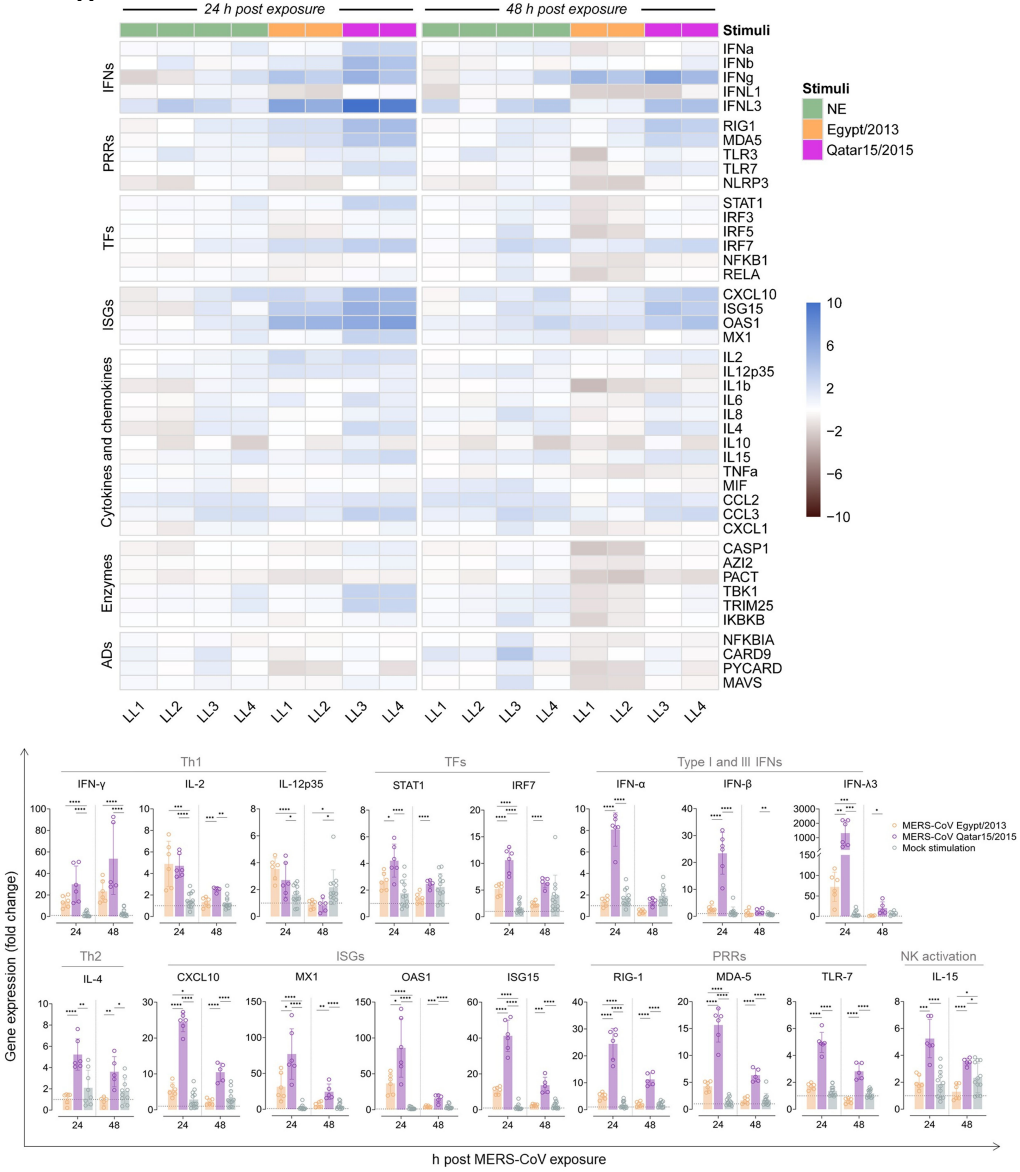
Expression of immune response genes by llama lymph node (LN) cells pulsed with MERS-CoV. A microfluidic RT-qPCR assay was used to quantify transcripts of immune-related genes at different h post MERS-CoV exposure (hpe). **(A)** After relative normalization, mean expression values (triplicates) of llama LN cells exposed for 24 and 48 h to MERS-CoV Qatar15/2015 (purple rectangles), Egypt/2013 (orange rectangles), or cultured in media only (NS, green rectangles) were calculated respective to non-cultured control cells. Mean log2 fold-change expression values of each studied gene are represented in a heat-map plot with colour variations; blue for up-regulated and black for down-regulated gene expression, respectively. Panel **(B)** display the relative expression values of some differentially regulated genes at 24 and 48 h after exposure to MERS-CoV Qatar15/2015 (purple), Egypt/2013 (orange) or cultured in media only (green). Boxes indicate mean expression values and error bars represent SD intervals. Individual relative expression measurements are shown as empty circles. Grey dashed lines display basal expression levels from freshly isolated control cells. *, *p*-value < 0.05; **, *p*-value < 0.001; ***, *p*-value < 0.0001; ****, *p*-value < 0.00001; ADs, adaptors; IFNs, interferons; ISGs, IFN stimulated genes; LL, llama; NK, natural killer T cells, PRRs, pattern-recognition receptors; TFs, transcription factors; Th1, T-helper 1; Th2, T-helper 2.

We also studied whether LN cells could mount immune responses to a secondary viral exposure *in vitro*. Transcriptomic profiles from 43 immune response genes were obtained using a previously described microfluidic RT-qPCR assay (35), which included the quantification of type I, II and III interferons (IFNs), pattern recognition receptors (PRRs), transcription factors (TFs), IFN-stimulated genes (ISGs), cytokines and chemokines involved in inflammatory responses, among other immune-related genes (**Figure 2A**). Afterwards, gene expression levels of LN cells exposed to MERS-CoV and mock-treated samples were compared to those from freshly isolated cells. Mock-exposed cells experienced a mild increase of immune response genes transcription and this was more evident at 48 h post *in vitro* culture (**Figure 2A, B**). *IFN-γ* expression was significantly up-regulated in MERS-CoV-treated cells and progressively increased over time (**Figure 2A, B**). Although not statistically significant, a stronger induction of *IFN-γ* occurred in cells exposed to the Qatar15/2015 strain than the ones exposed to the Egypt/2013 (**Figure 2B**). Expression levels of *IL-2* and *IL-12* similarly increased at 24 hpe and subsequently returned to basal levels (**Figure 2B**). On the other hand, an increase of *IL-4* expression was observed in cells exposed to the MERS-CoV Qatar15/2015 but not to Egypt/2013 strain (**Figure 2A, B**). Remarkably, induction of *IL-10* mRNA was not detected in any llama cells. Overall, results are reminiscent of a Th1 response elicited in LN cells after re-exposure to MERS-CoV, regardless of the strain used for stimulation.

Innate immune gene responses were also monitored. Transcription of *IFN-λ3* was markedly upregulated in cells treated with both MERS-CoV strains, being significantly higher in those stimulated with the Qatar15/2015 strain (**Figure 2B**). However, type I IFNs (*IFN-α* and *IFN-β*) were only upregulated in LN cells exposed to the Qatar15/2015 strain at 24 hpe (**Figure 2B**). Expression of TFs (*STAT1* and *IRF7*), ISGs (*CXCL10*, *MX1*, *OAS1* and *ISG15*), and PRRs (*RIG-1*, *MDA-5* and *TLR-7*) were enhanced in cells according to levels of IFNs (**Figure 2A, B**). Thus, the Egypt/2013 strain moderately induced the above-mentioned genes at 24 hpe, while higher upregulations occurred in cells exposed to the Qatar15/2015 strain that waned over time for both strains (**Figure 2B**). In addition, a significant mild upregulation of *TRIM25*, *CCL3*, and *IL-15* was mostly observed at 24 hpe by cells exposed to the Qatar15/2015 strain (**Figure 2A**). Importantly, pro-inflammatory responses were not induced along the study. These results evidenced that early and transient antiviral cellular immune responses were effectively triggered in LNs of llamas re-exposed to MERS-CoV. Responses induced by the MERS-CoV Qatar/2015 strain were significantly more pronounced than those provoked by the Egypt/2013 strain.

## Discussion

The present study suggests that cervical LN cells from llamas do not support MERS-CoV replication *in vitro*. In agreement, we previously described low levels of dipeptidyl-peptidase 4 (DPP4) receptor in lymphoid cells of camelids compared to other species (30, 39). Therefore, we employed an MOI of 0.1 to maximize the probabilities of LN cells to interact with MERS-CoV. The absence of an increase in genomic and subgenomic viral RNA over time supports that MERS-CoV replication did not occur in cultured cells. Furthermore, the expression of DPP4 and MERS-CoV nucleoprotein did not co-localize in llama cervical LNs *in vivo* (30). There is no data on the replication of MERS-CoV in LNs of other susceptible species. Nonetheless, our findings support the concept that camelid dendritic-like cells carry MERS-CoV to LNs without active viral replication, and they might be the drivers of potent immune responses that prevent virus spread.

We investigated if llamas could mount cellular adaptive immune responses to counteract MERS-CoV infection. Indeed, LN cells of previously infected llamas and subsequently exposed to the virus *in vitro* developed an early induction of *IL-12* in all MERS-CoV pulsed cells, suggesting that both Qatar15/2015 and Egypt/2013 strains were effectively mounting an immune response accompanied by an increase of *IFN-γ* over time. The concomitant induction of *IL-2* suggested activation of Th1 lymphocytes, similar to previous findings in PBMCs from convalescent human patients pulsed with MERS-CoV peptide pools (6, 10). Alternatively, or in addition, NK cells residing in camelid LN could be responsible for the up-regulation of *IFN-γ*, as previously described in cattle and human (40, 41). On the other hand, absence of *IL-10* up-regulation would indicate that Th2 cells were not induced or recalled. Further detailed studies are needed to deeply characterize T- and B- cell responses in LNs.

Importantly, significant induction of type I, II and III IFNs was noticed in LN cells of animals primed and re-stimulated *in vitro* with at least the Qatar15/2015 isolate, with a consequent up-regulation of ISGs, PRRs and TFs involved in antiviral responses. Strikingly, pro-inflammatory cytokines (*TNF-α*, *IL-1β*, *IL-6* and *IL-8*), *CARD9* (an activator of NF-κB) and components of the inflammasome (*NLRP3*, *CASP1*, *PYCARD*) remained at basal transcription levels or were slightly up- or down-regulated. Differences in these pro-inflammatory cytokines would be expected between species, as humans can suffer from severe MERS but camelids are asymptomatic hosts. This would imply specific mechanisms of camelids for dampening inflammation as observed in bats (24, 25). In these virus-tolerant animals, NF-κB-dependent inflammatory genes are inhibited under the action of C-Rel (24). Similar studies should be performed in camelid species to precisely determine mechanisms controlling inflammation and their similarity to those engaged in bats. Nonetheless, as bats, camelids can control inflammation mediating an impaired NLRP3 inflammasome. In the present study, *IFN-λ3* but not *IFN-λ1* was highly up-regulated and might contribute to counterbalance the inflammatory effects of type I IFNs (42). Moreover, control of inflammation is not specific to LN cells, since we previously described dampened inflammatory responses in the nose, trachea, and lungs of MERS-CoV-challenged alpacas. Early and transient type I and III IFNs were also produced by the nasal epithelium of these animals (22, 23). A previous study unravelled the high production of type I and III IFNs by human plasmacytoid DCs (pDCs) in the absence of productive MERS-CoV replication (43). We hypothesize that camelid pDCs sensing by MERS-CoV could be the source of the pronounced *IFN-λ3* response in LNs. Altogether, our results highlight that *IFN-λ3* might have a key role in bridging innate and adaptive immunity from the infected respiratory mucosa to secondary lymphoid organs, as previously described for other viral infections (44, 45). Thus, camelid species own key mechanisms to host MERS-CoV in the absence of clinical disease.

At 24 hpe, the Qatar15/2015 strain induced higher antiviral transcripts than the Egypt/2013 strain, while levels of cytokine mRNAs decayed thereafter except for *IFN-γ*. Possibly, pathogen-associated molecular patterns (PAMPs) of the Qatar15/2015 strain better activated type I and III IFN pathways. Alternatively, the peak of antiviral responses could be elicited earlier with the Egypt/2013 strain. However, our observations should be confirmed with samples from a larger number of animals, being also collected at early time points after viral exposure. Overall, llama cervical LN cells elicited early antiviral responses in the absence of inflammation to MERS-CoV re-exposure, which were higher for the clade B strain compared to its clade C counterpart.

A potential limitation of our study is the lack of comparison with LN cells from healthy, non-convalescent animals, which may help to discern unique features of camelid memory T-cell responses *versus* those occurring in a primary infection. Finally, the use of peptide pools to stimulate camelid LN lymphocytes would reveal the most immunogenic MERS-CoV-specific T cell epitopes, and thus, improve animal vaccine design.

In conclusion, the present study suggests that camelid LN cells could not support MERS-CoV replication. Remarkably, convalescent llamas developed strong cellular antiviral responses that are rapidly activated *in vitro* following a secondary viral exposure, in the absence of inflammation.

## Data availability statement

The original contributions presented in the study are included in the article/**Supplementary Material**. Further inquiries can be directed to the corresponding author.

## Ethics statement

The animal study was reviewed and approved by Ethical and Animal Welfare Committee of IRTA (CEEA-IRTA) and by the Ethical Commission of Animal Experimentation of the Autonomous Government of Catalonia (file No. CEA-OH/10942/1).

## Author contributions

JR, JS, JV-A and AB conceived and designed the study. JR, NT, JS, JV-A and AB performed the experiments and analyzed the data. All the authors discussed the results. The manuscript was written by JR and AB. All the authors revised the manuscript. All authors contributed to the article and approved the submitted version.
