# Supplementary figures and images for: Enhanced antiviral immunity and dampened inflammation in llama lymph nodes upon MERS-CoV sensing: bridging innate and adaptive cellular immune responses in camelid reservoirs
Source: Front Immunol. 2023 Jun 14;14:1205080. doi: 10.3389/fimmu.2023.1205080 (PMC10300347; doi:10.3389/fimmu.2023.1205080)

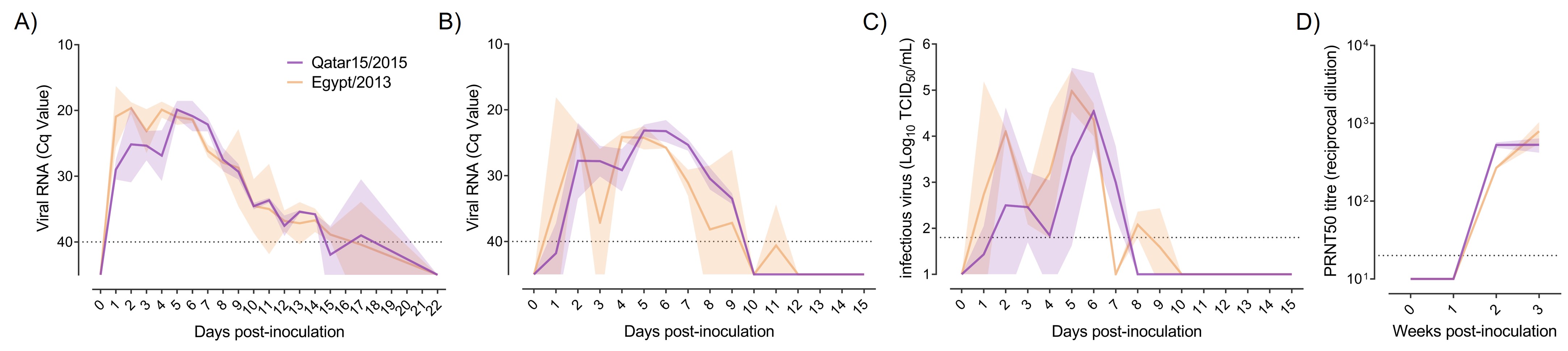

Supplement: Supplementary file 1 [file Image_1.jpeg]
